# MicroRNAs: Emerging players in the pathogenesis of vitiligo

**DOI:** 10.3389/fcell.2022.964982

**Published:** 2022-09-16

**Authors:** Xin Yu, Yuli Cui, Xueqing Zhu, Hongjun Xu, Linfeng Li, Guangcheng Gao

**Affiliations:** Department of Dermatology, Beijing Friendship Hospital, Capital Medical University, Beijing, China

**Keywords:** MicroRNAs, vitiligo, pathogenesis, miRNA, MiR-25

## Abstract

Vitiligo is an autoimmune skin disease characterized by presence of pale patchy areas of depigmentation. MicroRNAs (miRNAs) are important regulators of gene expression and play significant roles in diverse biological and pathological processes. Accumulating evidence has shown that miRNAs were differentially expressed in skin lesions and peripheral blood mononuclear cells of patients with vitiligo. In particular, miRNAs are significantly correlated with the development and progression of vitiligo. The abundance of some miRNAs in serum was also correlated with the vitiligo lesion severity, indicating that miRNAs might serve as prognostic biomarkers. Importantly, the direct involvement of miRNAs in the pathogenesis of vitiligo has been demonstrated. For example, increased expression of miR-25 contributes to vitiligo through promoting the dysfunction and oxidative stress-induced destruction of melanocytes. However, there are limited studies on the function and mechanism of deregulated miRNAs in vitiligo. Further studies are required to establish clinical applications of miRNAs for vitiligo. More in-depth investigations of miRNAs are needed for the understanding of the pathogenesis of vitiligo and the development of novel therapeutic targets. This present review summarizes the current literature on the deregulation and pathogenic roles of miRNAs in vitiligo. We also highlight the potential clinical applications of miRNAs in patients with vitiligo.

## Introduction

Vitiligo is a common refractory autoimmune skin disease characterized by presence of pale patchy areas of depigmentation with regular borders and sharp margins (Taieb and Picardo, 2009; Ezzedine et al., 2012). The depigmentation in vitiligo is caused by aberrant immune destruction of melanocytes from the epidermis and the follicular reservoir in the skin (Jin et al., 2016). It has been estimated that the incidence of vitiligo is approximately 0.5–1% worldwide with 37% of patients diagnosed before the age of 12 ((Ezzedine et al., 2012), (Boisseau-Garsaud et al., 2000)). Vitiligo negatively affects the quality of patient life and leads to serious emotional stress (Boniface et al., 2017; Vachiramon et al., 2017). Although various strategies including topical steroids, calcineurin inhibitors, vitamin D derivatives, phototherapy, excimer laser and surgical techniques have been devised for the treatment of vitiligo, an effective approach that ensures a complete cure is still lacking (Whitton et al., 2015; Iannella et al., 2016; Whitton et al., 2016). Both genetic and environmental factors play important roles in the pathogenesis of vitiligo (Kim et al., 1998). However, the exact etiology of vitiligo remains unknown (Schallreuter et al., 2008; Mohammed et al., 2015). Multiple hypothetic theories have been developed to explain the pathogenesis of vitiligo. Among them, the autoimmune hypothesis is the most accepted one.

MicroRNAs (miRNAs) are a class of small (approximately 22 nucleotides) non-coding RNAs that regulate gene expression at the post-transcriptional level. MiRNAs exert their functions by binding to the 3′-untranslated region (UTR) of their target messenger RNAs (mRNAs), which results in mRNA degradation or translation inhibition of their targets. Approximately 60% of protein-coding genes in the human genome are predicted to be regulated by miRNAs. Given their pervasive role in the regulation of gene expression, miRNAs have been found to play significant roles in different biological and pathological processes, including cell differentiation, proliferation and apoptosis, in human. Recent studies have shown that miRNAs could regulate the development and function of immune cells and melanocytes and participate in the pathogenesis of vitiligo (Mansuri et al., 2016; Šahmatova et al., 2016; Shang and Li, 2017).

In this review, we summarize the current literature on the deregulation and pathogenic roles of miRNAs in vitiligo. We would also highlight the potential clinical applications of miRNAs for the prognostication and treatment of patients with vitiligo^1^.

## Differentially expressed miRNAs in vitiligotbl1

### Altered miRNA expression in vitiligo serum

Table 1 Shi and others demonstrated that the levels of 20 miRNAs in serum were altered in mice with autoimmune vitiligo as compared with the control mice (Shi et al., 2014). TaqMan-basedreverse-transcription (RT)-quantitative PCR further confirmed the increased abundance of three miRNAs, namely miR-146a, miR-191, and miR-342-3p. Interestingly, these vitiligo-associated miRNAs have been reported to regulate the function of immune cells or proliferation and apoptosis of melanocytes. For instance, a previous study showed that miR-146 played a crucial role in the prolonged production of tumor necrosis factor (TNF)-α and significantly upregulated phagocytic activity of Natural Killer (NK) T cells (Pauley et al., 2008). TNF-α and NKT cells are involved in the pathogenesis of non-segmental vitiligo (NSV) whereas TNF-α inhibitors have been reported to successfully treat cases of vitiligo (Campanati et al., 2010; Kim et al., 2011). Therefore, elevated levels of serum miR-146a might take part in the pathogenesis of vitiligo. In addition, miR-191 was reported to modulate cell proliferation and survival of melanocytes and melanoma cells (Mueller et al., 2009).

**TABLE 1:** miRNAs expression profiles in vitiligo.

	Method	Sample	Up	Down	Ref
1	Microarray RT-PCR	Serum of vitiligo mice	3 miRNAs: miR-146a, miR-191, miR-342-3p		28
2	Microarray RT-PCR	Serum of NSV patients	17 miRNAs: miR-16, miR-451, miR-223, miR-19b, miR-151-5p, miR-25, miR-186, miR-195, miR-590-5p, miR-19a, miR-30days, miR-192, miR-222, miR-93, miR-146a, miR-29a, miR-191	14 miRNAs miR-1274A, miR-574-3p, miR-1290, miR-720,miR-10a, miR-483-5p, miR-125b, miR-150, miR-139-5p, miR-26a, let-7days, miR-30b,miR-30c, miR-126	33
3	RT-PCR	Skin lesion from aNSGV vs tNSGV	7 miRNAs miR-10a, miR-16, miR-125a, miR-139, miR-145, miR-191, miR-574	miR-21, miR-31, miR-192	34
		PBMCs of NSGV patient vs. healthy controls	12 miRNAs: miR-10a, miR-16, miR-21, miR-31, miR-125a, miR-139, miR-145, miR-146a, miR-181a1, miR-191, miR-192, miR-203		
4	Microarray RT-PCR	Skin lesions of NSV patients compared with healthy skin from controls	12 miRNAs: miR-1, miR-133b, miR-135a miR-183, miR-190, miR-214, miR-301b, miR-30a-3p, miR-375, miR-487a, miR-517c, miR-616	1 miRNAs miR-211	35
		Skin lesions of NSV patients compared with nonlesional skin		miR-136, miR- 296 and miR-328	
5	Microarray	Lesional epidermal skin in vitiligo patients	56 miRNAs		36
6	Microarray RT-PCR	PBMCs of NSV patients	2 miRNAs: miR-224-3p, miR-4712-3p	1 miRNAs miR-3940-5p	37
7	RNA-seq	PBMCs of NSV patients	223 miRNAs, Top 10: miR-335-5p, miR-20a-5p, miR-514a-3p, miR-144-5p, miR-450b-5p, miR-369-3p, miR-101-3p, miR-142-5p, miR-19b-3p, and miR-340-5p	100 miRNAs, Top 10: miR-4443, miR-1248, miR-6859-3p, miR-668-3p, miR-7704, miR-323a-5p, miR- 1237-3p, miR-3127-3p, miR-6735-3p, and miR-127-3p	38

Another study showed that the levels of 31 miRNAs in serum were significantly different between patients with NSV and healthy controls (Ling et al., 2013), among which 12 miRNAs changed greater than three folds. In particular, serum miR-16 and miR-19b were upgulated and miR-720 was downregulated in vitiligo, serving as the best marker in differentiating NSV from healthy controls. Moreover, the serum level of miR-574-3p was correlated with the lesion severity of NSV. These two studies collectively demonstrated that serum miRNAs might take part in the pathogenesis of NSV and could serve as potential biomarkers for its prognosis. However, further investigations are required to validate the clinical usefulness of serum miRNAs in larger cohorts of NSV patients from different populations.

Parihar et al. investigated the expression of miRNA and pigmentation associated genes in active non-segmental generalized vitiligo (aNSGV) (Parihar et al., 2020). The expression of miR-16 and miR-145 were significantly higher in all three compartments i.e. PBMCs, serum and lesional skin of aNSGV than treated aNSGV group. The expression of miR-10a, miR-16, miR-21, miR-31, miR-125a, miR-139, miR-145, miR-146a, miR-181a1, miR-191, miR-192 and miR-203 was significantly upregulated in PBMCs of aNSGV compared to healthy controls. In addition, the expression of miR-10a, miR-16, miR-145, miR-146a, miR-155 and miR-574 was downregulated in PBMCs of treated aNSGV compared to aNSGV. The expression of miR-16, miR-145 and miR-203 was positively correlated for in PBMCs of aNSGV with BSA% affected. MiR-574 expression was negatively correlated with BSA% repigmentation in treated aNSGV group.

### Altered miRNA expression in vitiligo skin

miRNA profiling was performed in skin lesions of patients with NSV. The profiles were compared with those of non-lesioned skin of NSV patients or healthy human skin (Mansuri et al., 2014). The expression of a total of 12 miRNAs, including miR-1, miR-9 and miR-135a, was significantly upregulated in NSV lesions compared with healthy skin. Previous studies showed that miR-9 and miR-135a could target the expression of SIRT1, which is known to protect against aging and other stress-related diseases, implicating that the circuitry formed between miR-9/135a and Sirtuin 1 (SIRT1) might be involved in the destruction of melanocytes. Conversely, miR-141 expression was downregulated in the non-lesioned skin of NSV patients as compared with healthy human skin, indicating that the microenvironment in non-lesioned skin of NSV patients is also different from that of the healthy controls.

To investigate effect of NB-UVB treatment on miRNA expression, Parihar et al. studied the expression of miRNA (peripheral blood, serum, lesional and nonlesional skin) in untreated active NSGV (aNSGV) and NB-UVB-treated aNSGV (tNSGV). The expression of miR-10a, miR-16, miR-19a, miR-21, miR-31, miR-125a, miR-139, miR-145, miR-191, miR-192 and miR-574 was significantly upregulated in lesional and non-lesional skin of aNSGV and treated aNSGV (22). However, miR-10a, miR-16, miR-125a, miR-139, miR-145, miR-191 and miR-574 expression was significantly downregulated in treated lesional compared to untreated lesional skin. Moreover, miR-145 expression was significantly positively correlated with BSA% affected in lesional skin of aNSGV. Among these miRNAs, miR-16 and miR-145 expression was reduced in all three compartments, i.e. PBMCs, serum and lesional skin in the tNSGV group compared with the aNSGV group.

Vaish et al. found a total of 56 upregulated miRNAs of more than 1.5 fold in the lesional epidermal skin in vitiligo. Of the 56 upregulated miRNAs, the expression levels of 29 miRNAs were significantly higher in the lesional epidermis than the non-lesional epidermis (Vaish et al., 2019).

### Altered miRNA expression in peripheral blood mononuclear cells

MiR-224-3p and miR-4712-3p expression was increased whereas miR-3940-5p expression was decreased in the peripheral blood mononuclear cells (PBMCs) isolated from NSV patients (Wang et al., 2015). Thymosin α1 is a naturally occurring polypeptide with immune-modulating functions and an excellent safety profile in the clinic when used as an adjuvant or an immunotherapeutic agent. It was found that thymosin α1 could abrogate the deregulation of these miRNA in PBMCs of NSV patients. Therefore, these results suggested that differentially expressed miRNAs in PBMCs may be involved in the immune imbalance in the development of vitiligo.

Another study also analyzed miRNA expression profile in the PBMCs of NSV patients (Zhang et al., 2020). The top 10 up-regulated miRNAs included miR-335-5p, miR-20a-5p, miR-514a-3p, miR-144-5p, miR-450b-5p, miR-369-3p, miR-101-3p, miR-142-5p, miR-19b-3p, and miR-340-5p. The top 10 down-regulated miRNAs were miR-4443, miR-1248, miR-6859-3p, miR-668-3p, miR-7704, miR-323a-5p, miR-1237-3p, miR-3127-3p, miR-6735-3p, and miR-127-3p. Moreover, miR-20a-5p expression was remarkably increased in PBMCs of progressive and stable NSV compared to the healthy controls. MiR-20a-5p can be a potential marker for stage assessment in PBMCs of NSV patients.

### Altered miRNA expression in exosomes

Exosomes are nano-sized extracellular vesicles released from most cell types into the extracellular environment. Exosomes contain functional proteins, including mRNAs and miRNAs, acting crucial roles in maintaining the internal balance. Circulating exosomal miRNAs may act as diagnostic and therapeutic biomarkers for skin diseases. A recent study high-throughput sequencing of exosomal miRNA expression profiles in serum from SV patients compared with healthy controls (Dong et al., 2022). Among 50 differentially expressed miRNAs, seven miRNAs were identified as biomarker candidates associated with SV. The expression of miR-493-3p, miR-370-3p, and miR-143-5p was increased. The expression of^2^ miR-885-5p, miR-16-5p, miR-92a-3p, and miR-92b-3p was reduced. MiR-200c was significantly down-regulated in exosomes released from keratinocytes in vitiligo patients (Zhao et al., 2020). MiR-493-3p expression is significantly increased in circulating exosomes and perilesions in patients with SV. Circulating exosomes were internalized by human primary keratinocytes and increase dopamine secretion *in vitro*.

## The role of miRNA in various pathogenesis processes of vitiligo

The etiology and pathogenesis of vitiligo remain unclear. Numerous factors have been proved to partipate in vitiligo development, including: genetic background, stress, trauma, infections, cancers, sunlight, neural and endocrine diseases, melanocyte and melatonin receptor dysfunction, drugs and cytotoxic compounds (Delmas and Larue, 2019). However, the mechanism underlying melanocyte destruction remains to be fully elucidated. It is widely accepted that ROS is the most crucial trigger, causing a small fraction of melanocytes deaths. These melanocyte deaths lead to antigens exposure and inflammation, which breaches self‐tolerance to melanocyte. Then, self‐responsive immune function finally leads to directly contributes to the majority of melanocyte deaths in vitiligo.

### Oxidative stress damage

Oxidative stress is a crucial initiator in vitiligo occurrence (Di Dalmazi et al., 2016). The oxidative stress hypothesis indicated the elevated production of reactive oxygen species (ROS), leading to immune response and melanocyte death (Xuan et al., 2022). ROS leads to a minority of melanocyte demise by causing molecular and organelle dysfunction, as well as melanocyte‐specific antigens exposure. Deregulated miRNAs may promote vitiligo development by regulating the expression and function of oxidative stress related genes in melanocytes. Approaches to ameliorate oxidative stress is an effective therapeutic strategy by preventing melanocytes from destruction.

miR-1 expression was significantly increased in NSV lesions. Previous reports showed that, the increased expression of miR-1 plays a potential role in the response to oxidative stress which favors melanocyte apoptosis. Oxidative stress increased miR-25 expression in both melanocytes and keratinocytes. MiR-25 could inhibit the production and secretion of growth factors stem cell factor and basic fibroblast growth factor from keratinocytes, protecting melanocytes survival under oxidative stress. MiR-135a and miR-9 expression significantly increased in NSV skin (Mansuri et al., 2014; Mansuri et al., 2016). MiR-135a and miR-9 are known to target SIRT1 expression, which may protect against stress-related diseases (Wu et al., 2019). rs11614913 C allele in miR-196a-2 protect against oxidative effects on human melanocytes through regulating its target gene *TYR*P1 (tyrosinase-related protein 1), decreasing the risk of vitiligo. Keratinocytes transfected with miR-493-3p increased ROS levels and decreased melanocyte proliferation and melanin synthesis (Dong et al., 2022).

### Immune imbalance

In Th17-polarized CD4^+^ T cells, miR-21-5p could reduce the proportion of effector T cells and associated cytokines while upregulate the proportion of regulatory T cells and Foxp3. Overexpression of miR-21-5p in Th17-polarized CD4^+^ T cells decreased apoptosis of melanocytes. miR-133b was upregulated in lesional skin from nonsegmental vitiligo through targeting IL17 A/F. In addition, IL-17A/F and IL-22 secretion from Th17 cells could increase production of chemokines and cytokines in vitiligo (Mansuri et al., 2014). MiR-155 expression could be induced by several vitiligo-associated cytokines. MiR-155 upregulation might regulate the inflammatory stimuli-induced MITF-M downregulation. Elevated miRNA-377 expression might contribute to the pathogenesis of vitiligo through PPAR-γ downregulation and IL-17 upregulation (Alhelf et al., 2022). MiR-3940-5p expression was decreased in PBMCs isolated from NSV patients (Wang et al., 2015). Previous study showed that miR-3940-5p downregulation promoted T-cell immnity by targeting the IL-2R, indicating a potential immune role of miR-3940-5p in vitiligo.

### Abnormal melanocyte function

**FIGURE 1:**
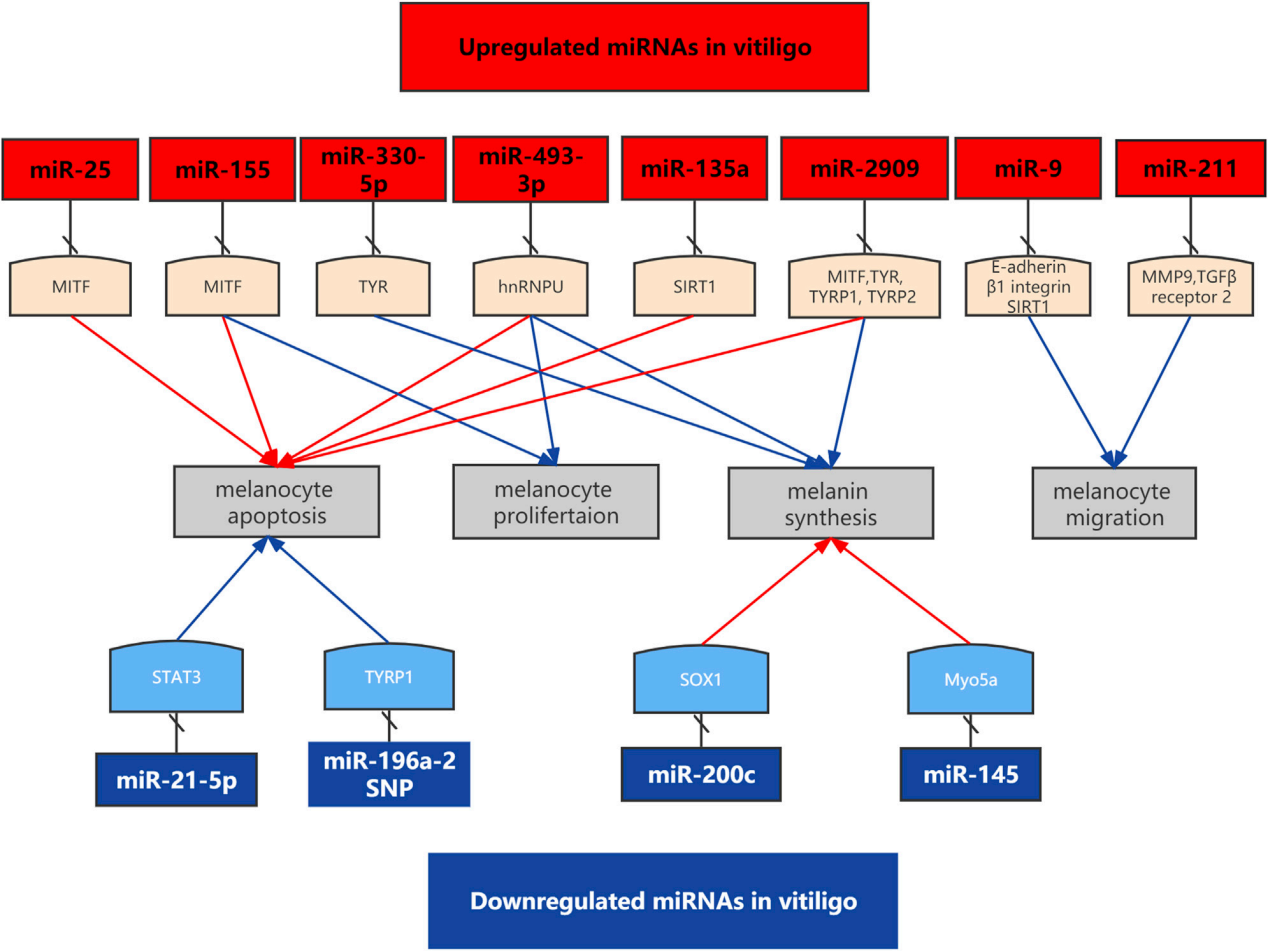
Functional roles of specific deregulated miRNAs in the cell apotosis, melanin synthesis and cell migration in vitiligo.

Figure 1 Melanocytes deaths may be caused by abnormal proliferation, differentiation, migration and apoptosis. Microphthalmia-associated transcription factor (MITF) is a master regulator in the survival and function of melanocytes. MiRNAs participate in the pathogenesis of vitiligo by regulating melanocytes function. MiR-9 suppressed migration of PIG1 cells to UVB exposed HaCaT cells through targeting E-cadherin and β1 integrin in HaCaT cells. Increased expression of miR-25 promoted the dysfunction and oxidative stress-induced destruction of melanocytes. Overexpression of miR-155 decreased the expression of melanogenesis-associated genes in melanocytes and keratinocytes (Šahmatova et al., 2016). MiR-211 inhibited UVB-induced human melanocyte migration *via* inhibiting MMP9 (matrix metalloprotease 9) and p53 (Dai et al., 2015). MiR-211 inhibited UVB-induced human melanocyte migration *via* inhibiting MMP9 (Sahoo et al., 2017). MiR-2909 inhibited melanin synthesis genes and induced melanocytes destruction in vitiligo^3^.

## Deregulated miRNAs of functional importance in vitiligo

### MiR-9

Table 2 Su et al. investigated the role and mechanism of miR-9 in melanocytes during vitiligo repigmentation after UVB exposure (Su et al., 2019). They used HaCaT keratinocytes to mimic vitiligo lesional condition and the PIG1 melanocytes as perilesional condition. MiR-9 expression was upregulated in human lesional vitiligo specimens. In addition, the expression of adhesion molecules such as E-cadherin and β1 integrin was decreased in human lesional vitiligo specimens. UVB exposure decreased miR-9 expression while increased the expression of IL-10, E-cadherin, and β1 integrin in HaCaT cells. Moreover, the increased IL-10 by UVB exposure decreased miR-9 level by promoting miR-9 methylation in HaCaT cells. MiR-9 suppressed migration of PIG1 cells to UVB exposed HaCaT cells through targeting E-cadherin and β1 integrin in HaCaT cells. In conclusion, miR-9 decreased the migration of PIG1 cells to HaCaT cells during UVB-induced repigmentation in vitiligo, providing new therapeutic targets for vitiligo.

**TABLE 2:** Functional characterization of the miRNAs in vitiligo.

miRNAs	Expression	Functional role	Related gene	Role	Ref
miR-9	Up	Inhibits migration of PIG1 cells to HaCaT	E-cadherin and β1 integrin	damage	38
miR-21-5p	Down	Inhibits melanocyte apoptosis	STAT3	protect	39
miR-25	Up	Promotes melanocyte degeneration	MITF	damage	41
miR-133	Up	-	IL17 A/F	-	23
miR-135a	Up	Promotes melanocyte destruction	SIRT1	damage	33
miR-145	Down	Perinuclear accumulation of melanosomes with hypopigmentation	Myo5a	protect	52
miR-155	Up	Inhibits melanocyte proliferation. Promotes apoptosis	MITF	damage	42, 43
MiR-183-5p	Up	-	MITF	-	53
miR-196a-2	Polymorphisms	Inhibits melanocyte apoptosis	TYRP1	protect	47
miR-200c	Down	Promotes melanin synthesis	SOX1	protect	28
miR-211	Down in UVB-treated melanocytes	Inhibits UVB-induced melanocyte migration	MMP9, SIRT1, TGF beta receptor 2	damage	36, 49
miR-330-50	Up	Inhibits melanin levels	TYR	damage	55
miR-377	Up	-	PPAR-γ	-	51
miR-493-3p	Up	Promotes melanocyte apoptosis, Inhibits melanocyte proliferation and melanin synthesis	hnRNPU	damage	27
miR-2909	Up	Inhibits melanin synthesis	MITF, TYR, TYRP1, TYRP2	damage	54

### MiR-21-5p

A recent study demonstrated that miR-21-5p played a protective role in the pathogenesis of vitiligo (Huo et al., 2021). miR-21-5p, also known as miR-21, was downregulated in patients with vitiligo. In Th17-polarized CD4^+^ T cells, miR-21-5p could reduce the proportion of effector T cells and associated cytokines while upregulate the proportion of regulatory T cells and Foxp3. Overexpression of miR-21-5p in Th17-polarized CD4^+^ T cells decreased apoptosis of melanocytes. Further experiment proved that signal transducer and activator of transcription 3 (STAT3) was the target gene of miR-21-5p. STAT3 expression was increased in patients with vitiligo and STAT3 could partly reverse the effects of miR-21-5p overexpression on melanocytes. To conclude, miR-21-5p may play a protective role in vitiligo via targeting STAT3. Another study measured serum levels of miR-21-5p in 40 NSV patients compared with 40 healthy controls. Serum miR-21-5p expression in vitiligo patients was higher compared with the healthy controls (Aguennouz et al., 2021). The relative miR-21-5p expression was directly correlated with VASI (Vitiligo Area and Severity Index) score.

### MiR-25

miR-25 expression was increased in both serum and skin lesion samples from vitiligo patients (Shi et al., 2016). In addition, serum level of miR-25 was positively correlated with the disease activity of vitiligo. Increased expression of miR-25 promoted the dysfunction and oxidative stress-induced destruction of melanocytes. Interestingly, MITF was found to be targeted by miR-25 and account for the miR-25-induced dysfunction and destruction of melanocytes. Oxidative stress increased miR-25 expression in both melanocytes and keratinocytes possibly by promoting demethylating of miR-25-encoding gene. Interestingly, miR-25 also inhibited the production and secretion of growth factors stem cell factor and basic fibroblast growth factor from keratinocytes, which could protect melanocytes survival under oxidative stress. These data suggested that oxidative stress-induced overexpression of miR-25 contributes to vitiligo through influencing the degeneration of melanocytes via a MITF-dependent pathway and weakening the paracrine protective effect of keratinocytes. Further investigations are needed to explore the possibility of targeting miR-25 as a therapeutic approach for vitiligo.

### MiR-155

miR-155 expression was upregulated in skin lesions from patients with vitiligo (Šahmatova et al., 2016). Interestingly, ectopic expression of miR-155 has been shown to impair proliferation and induces apoptosis of melanoma cells. Overexpression of miR-155 also decreased the expression of melanogenesis-associated genes in melanocytes and keratinocytes. In addition, miR-155 expression could be induced by several vitiligo-associated cytokines. Regarding the downstream mechanism, a recent study demonstrated that miR-155 was able to target endogenous MITF in melanoma cells. The role for miR-155 in the interleukin-1β-induced repression of MITF was also established (Arts et al., 2015). In summary, these findings suggested that miR-155 might play a significant role in the pathogenesis of vitiligo.

### MiR-196a-2

Single-nucleotide polymorphism (SNP) is a common type of genetic variation in human (Zhao et al., 2017). It has been shown that SNPs in the pre-miRNA genes play crucial role in many physiological and pathological processes through regulating the expression and targeted selection of miRNAs (Fan et al., 2017; Wu et al., 2017). Huang and others demonstrated that the rs11614913 miR-196a-2 CC genotype was associated with a lower risk of vitiligo (Huang et al., 2013). TYRP1 is an enzyme in melanocytes, which promotes reactive oxygen species (ROS) formation by using an oxidative inducer as a substrate to catalyze quinone production. *TYRP1* gene expression was decreased by the rs11614913 C allele in miR-196a-2, thus decreasing the expression of intracellular ROS in melanocytes. Moreover, rs11614913 C allele in miR-196a-2 could reduce H_2_O_2_-induced apoptosis in melanocytes, indicating that the rs11614913 C allele in miR-196a-2 may decrease the risk of vitiligo and protect against oxidative effects on human melanocytes through regulating its target gene *TYRP1*. Another study showed that miR-196a-2 rs11614913 polymorphisms may regulate the expression levels of tyrosinase (Cui et al., 2015), which can control the production of melanin and influence the susceptibility of vitiligo. In this regard, the rs11614913 C allele in miR-196a-2 increased its regulatory effect on tyrosinase downregulation, which reduced melanocyte apoptosis rate and intracellular ROS levels. Taken together, rs11614913 polymorphism of miR-196a-2 is associated with the development of vitiligo by affecting the expression of Tyrp1 and tyrosinase.

### MiR-211

UVB-based phototherapy is an effective therapeutic option to improve repigmentation in vitiligo patients. SU et al. proved that p53-TRPM1/miR-211-MMP9 axis was activated and acted a central role in melanocyte migration (Su et al., 2020). The expression of p53 and MMP9 in melanocytes was upregulated in a dose-dependent manner after UVB exposures. MiR-211 expression level was inversely associated with MMP9 in the cells following UVB exposures. Moreover, miR-211 inhibited UVB-induced melanocyte migration via inhibiting MMP9. P53 directly reversed the inhibitory effect of miR-211 on melanocyte migration. Therefore, p53-TRPM1/miR-211-MMP9 axis serves as a potential target for repigmentation in vitiligo patients. Another study found MALAT1-miR-211-SIRT1 signaling axis potentially played protective role in the ‘amelanotic’ keratinocytes in vitiligo. Sirtuin1 (SIRT1) was proved to be a direct target of miR-211. Moreover, lncRNA MALAT1 was a negative upstream regulator of miR-211 (Brahmbhatt et al., 2021). Another study by Dai et al. showed deregulated miR-211 is implicated in melanomagenesis. MiR-211 expression positively regulates pigmentation by targeting TGF beta receptor 2 (Dai et al., 2015)^4^.

### MiRNA-377

MiRNA-377 is a conserved noncoding RNA that regulates angiogenesis and promotes oxidative stress. Peroxisome proliferator-activated receptors (PPARs), a member of the nuclear hormone receptor superfamily, play a key role in melanogenesis. A recent study showed that miRNA-377 and IL-17 expression was significantly elevated in the vitiligo group compared with the healthy control group. However, PPAR-γ and lncRNA TUG1 were significantly downregulated in vitiligo patients. To conclude, dysregulated miRNA-377 might contribute to the pathogenesis of vitiligo through PPAR-γ downregulation and IL-17 upregulation (Alhelf et al., 2021).

### miR-493-3p

A recent study showed that miR-493-3p/hnRNPU/COMT/dopamine axis may contribute to vitiligo development by affecting melanocyte function (Dong et al., 2022). Circulating exosomal miR-493-3p expression is significantly increased in circulating exosomes in patients with segmental vitiligo. MiR-493-3p overexpression in keratinocytes increased ROS levels and induced melanocyte apoptosis as well as decreased melanocyte proliferation and melanin synthesis. Heterogeneous nuclear ribonucleoprotein U (hnRNPU) was identified as a target of miR-493-3p.

### Other miRNAs

MiR-133b was upregulated in lesional skin from nonsegmental vitiligo through targeting IL17 A/F. In addition, IL-17A/F and IL-22 secretion from Th17 cells could increase production of chemokines and cytokines in vitiligo (Mansuri et al., 2014). MiR-145 was significantly downregulated in treated melan-a cells compared with untreated cells. MiR-145 expression in melan-cells was inversely correlated with expression of Sox9, Mitf, Tyr, Trp1, Myo5a, Rab27a, and Fscn1. Myo5a was a direct target of miR-145. Human melanocytes transfected with miR-145 showed perinuclear accumulation of melanosomes with hypopigmentation of harvested cell pellets (Dynoodt et al., 2013). MiR-183-5p expression was upregulated in depigmentation in black mice induced by an autoimmune response. They proved miR-183-5p as a direct regulator of MITF in melanocytes. MiR-183-5p is an important regulator of melanocytes function (Al Robaee et al., 2022). MiR-200c was significantly down-regulated in exosomes released from keratinocytes in vitiligo patients (Zhao et al., 2020). MiR-200c could increase the expression of melanogenesis related genes via suppressing SOX1 to activate β-catenin. In conclusion, keratinocyte-derived exosomal miR-200c played a key role in melanogenesis in vitiligo lesions. Therefore, it may be a potential target for the treatment of vitiligo. MiR-2909 expression was significantly elevated in skin of vitiligo patients compared to the healthy counterparts (Kaushik et al., 2020). Monobenzyl Ether of Hydroquinone (MBEH) could create pathophysiological manifestation similar to vitiligo. Ectopic cellular miR-2909 expression inhibited synthesis of skin melanin through inhibiting expression of critical melanin synthesis genes. In summary, miR-2909 might promote the initiation and development of vitiligo by inhibiting melanin synthesis genes and inducing melanocytes destruction. Overexpression of miR-330-5p inhibits melanin levels but not affect melanocyte function. Therefore, miR-330-5p induces depigmentation through targeting tyrosinase (TYR) (Rambow et al., 2014). Serum miR-720 was significantly downregualted in patients with NSV. Moreover, MITF knockdown may decrease the expression of miR-720 (Wang et al., 2012).

## Conclusion and future perspectives

Review of the literature indicates that miRNA is significantly correlated with progression and severity of vitiligo. Although the pathogenesis of vitiligo remains unclear, it is proved that miRNAs play a key role in the pathological process. These miRNAs participate in the process of oxidative stress reactions, immune imbalance and melanocyte dysfunction. Expression of particular miRNA was different in serum, PBMCs and skin lesions of patients with vitiligo. In addition, the levels of some serum miRNAs are correlated with the lesion severity of vitiligo, indicating that miRNA might serve as a prognostic biomarker. Importantly, miRNA may directly contribute to the pathogenesis of vitiligo. In particular, altered expression of miRNAs has significant effects on intracellular signaling network and thereby promoting degeneration of melanocytes in the development and progression of vitiligo. Future investigation is needed to discover more novel miRNA implicated in the pathogenesis of vitiligo.

Therapeutic applications based on miRNAs are highly promising. miRNA antagonists replacement represents a promising therapeutic strategy to gain repigmentation in vitiligo patient. Further studies are required to understand the upstream and downstream mechanisms and functional consequences of miRNA deregulation in vitiligo and establish the clinical usefulness of miRNAs for vitiligo management in clinical settings.

## Footnotes

1 MiRNAs networks involved in melanocyte function in vitiligo.

2 Upregulated miRNAs in vitiligo are listed in red text box while downregualted miRNAs are listed in blue text box.

3 Represenst the effects of activating downstream target genes, which interferes their expression.

4 Red arrows represent that miRNAs play the effects of promotion, and blue arrows represent miRNAs play the effects of suppression.

## Abbreviations

MITF, microphthalmia-associated transcription factor; TYR, tyrosinase; hnRNPU, heterogeneous nuclear ribonucleoprotein U; SIRT1, Sirtuin 1; TRP1, tyrosinase-related protein 1; MMP9, matrix metalloprotease 9; TGF-β receptor 2, transforming growth factor beta receptor 2; STAT3, signal transducer and activator of transcription 3; SOX1, SRY-box transcription factor 1; Myo5a, myosin VA.

## References

[B1] Aguennouz M. Guarneri F. Oteri R. Polito F. Giuffrida R. Cannavo S. P. (2021). Serum levels of miRNA-21-5p in vitiligo patients and effects of miRNA-21-5p on SOX5, beta-catenin, CDK2 and MITF protein expression in normal human melanocytes. J. Dermatol. Sci. 101 (1), 22–29. 10.1016/j.jdermsci.2020.10.014 33176966

[B2] Al Robaee A. A. Alzolibani A. A. Rasheed Z. (2022). MicroRNA-183-5p regulates MITF expression in vitiligo skin depigmentation. Nucleosides Nucleotides Nucleic Acids, 1–21. Online ahead of print. 10.1080/15257770.2022.2066126 35442159

[B3] Alhelf M. Rashed L. A. Ragab N. Elmasry M. F. (2021). Association between long noncoding RNA taurine-upregulated gene 1 and microRNA-377 in vitiligo. Int. J. Dermatol. 61, 199–207. 10.1111/ijd.15669 34014568

[B4] Alhelf M. Rashed L. A. Ragab N. Elmasry M. F. (2022). Association between long noncoding RNA taurine-upregulated gene 1 and microRNA-377 in vitiligo. Int. J. Dermatol. 61 (2), 199–207. 10.1111/ijd.15669 34014568

[B5] Arts N. Cané S. Hennequart M. Lamy J. Bommer G. Van den Eynde B. (2015). microRNA-155, induced by interleukin-1ß, represses the expression of microphthalmia-associated transcription factor (MITF-M) in melanoma cells. PLoS One 10 (4), e0122517. 10.1371/journal.pone.0122517 25853464 PMC4390329

[B6] Boisseau-Garsaud A. M. Garsaud P. Cales-Quist D. Helenon R. Queneherve C. Claire R. C. (2000). Epidemiology of vitiligo in the French west indies (isle of Martinique). Int. J. Dermatol. 39 (1), 18–20. 10.1046/j.1365-4362.2000.00880.x 10651958

[B7] Boniface K. Jacquemin C. Darrigade A. Dessarthe B. Martins C. Boukhedouni N. (2017). Vitiligo skin is imprinted with resident memory CD8 T cells expressing CXCR3. J. Invest. Dermatol. 138, 355–364. 10.1016/j.jid.2017.08.038 28927891

[B8] Brahmbhatt H. D. Gupta R. Gupta A. Rastogi S. Misri R. Mobeen A. (2021). The long noncoding RNA MALAT1 suppresses miR-211 to confer protection from ultraviolet-mediated DNA damage in vitiligo epidermis by upregulating sirtuin 1. Br. J. Dermatol. 184 (6), 1132–1142. 10.1111/bjd.19666 33152110

[B9] Campanati A. Giuliodori K. Ganzetti G. Liberati G. Offidani A. M. (2010). A patient with psoriasis and vitiligo treated with etanercept. Am. J. Clin. Dermatol. 11 (1), 46–48. 10.2165/1153424-S0-000000000-00000 20586509

[B10] Cui T. Yi X. Zhang W. Wei C. Zhou F. Jian Z. (2015). miR-196a-2 rs11614913 polymorphism is associated with vitiligo by affecting heterodimeric molecular complexes of Tyr and Tyrp1. Arch. Dermatol. Res. 307 (8), 683–692. 10.1007/s00403-015-1563-1 25896941

[B11] Dai X. Rao C. Li H. Chen Y. Fan L. Geng H. (2015). Regulation of pigmentation by microRNAs: MITF-dependent microRNA-211 targets TGF-beta receptor 2. Pigment. Cell Melanoma Res. 28 (2), 217–222. 10.1111/pcmr.12334 25444235

[B12] Delmas V. Larue L. (2019). Molecular and cellular basis of depigmentation in vitiligo patients. Exp. Dermatol. 28 (6), 662–666. 10.1111/exd.13858 30536790

[B13] Di Dalmazi G. Hirshberg J. Lyle D. Freij J. B. Caturegli P. (2016). Reactive oxygen species in organ-specific autoimmunity. Auto. Immun. Highlights 7 (1), 11. 10.1007/s13317-016-0083-0 27491295 PMC4974204

[B14] Dong L. Zhou T. She Q. Nie X. Liu Z. Pan R. (2022). Circulating exosomal miR-493-3p affects melanocyte survival and function by regulating epidermal dopamine concentration in segmental vitiligo. J. Invest. Dermatol. S0022-202X, 01529. 10.1016/j.jid.2022.05.1086 35690140

[B15] Dynoodt P. Mestdagh P. Van Peer G. Vandesompele J. Goossens K. Peelman L. J. (2013). Identification of miR-145 as a key regulator of the pigmentary process. J. Invest. Dermatol. 133 (1), 201–209. 10.1038/jid.2012.266 22895360

[B16] Ezzedine K. Lim H. W. Suzuki T. Katayama I. Hamzavi I. Lan C. C. (2012). Revised classification/nomenclature of vitiligo and related issues: The vitiligo global issues consensus conference. Pigment. Cell Melanoma Res. 25 (3), E1–E13. 10.1111/j.1755-148X.2012.00997.x PMC3511780 22417114

[B17] Fan L. Chen L. Ni X. Guo S. Zhou Y. Wang C. (2017). Genetic variant of miR-4293 rs12220909 is associated with susceptibility to non-small cell lung cancer in a Chinese Han population. PLoS One 12 (4), e0175666. 10.1371/journal.pone.0175666 28410417 PMC5391943

[B18] Huang Y. Yi X. Jian Z. Wei C. Li S. Cai C. (2013). A single-nucleotide polymorphism of miR-196a-2 and vitiligo: An association study and functional analysis in a han Chinese population. Pigment. Cell Melanoma Res. 26 (3), 338–347. 10.1111/pcmr.12081 23433405

[B19] Huo J. Liu T. Li F. Song X. Hou X. (2021). MicroRNA215p protects melanocytes via targeting STAT3 and modulating Treg/Teff balance to alleviate vitiligo. Mol. Med. Rep. 23 (1), 51. 10.3892/mmr.2020.11689 33200798 PMC7716409

[B20] Iannella G. Greco A. Didona D. Didona B. Granata G. Manno A. (2016). Vitiligo: Pathogenesis, clinical variants and treatment approaches. Autoimmun. Rev. 15 (4), 335–343. 10.1016/j.autrev.2015.12.006 26724277

[B21] Jin Y. Andersen G. Yorgov D. Ferrara T. Ben S. Brownson K. (2016). Genome-wide association studies of autoimmune vitiligo identify 23 new risk loci and highlight key pathways and regulatory variants. Nat. Genet. 48 (11), 1418–1424. 10.1038/ng.3680 27723757 PMC5120758

[B22] Kaushik H. Kaul D. Kumaran M. S. Parsad D. (2020). Chemical induced pathognomonic features observed in human vitiligo are mediated through miR-2909 RNomics pathway. J. Dermatol. Sci. 100, 92–98. 10.1016/j.jdermsci.2020.06.004 33039241

[B23] Kim N. H. Torchia D. Rouhani P. Roberts B. Romanelli P. (2011). Tumor necrosis factor-alpha in vitiligo: Direct correlation between tissue levels and clinical parameters. Cutan. Ocul. Toxicol. 30 (3), 225–227. 10.3109/15569527.2011.560913 21388239

[B24] Kim S. M. Chung H. S. Hann S. K. (1998). The genetics of vitiligo in Korean patients. Int. J. Dermatol. 37 (12), 908–910. 10.1046/j.1365-4362.1998.00549.x 9888330

[B25] Ling H. Spizzo R. Atlasi Y. Nicoloso M. Shimizu M. Redis R. S. (2013). CCAT2, a novel noncoding RNA mapping to 8q24, underlies metastatic progression and chromosomal instability in colon cancer. Genome Res. 23 (9), 1446–1461. 10.1101/gr.152942.112 23796952 PMC3759721

[B26] Mansuri M. Singh M. Begum R. (2016). miRNA signatures and transcriptional regulation of their target genes in vitiligo. J. Dermatol. Sci. 84 (1), 50–58. 10.1016/j.jdermsci.2016.07.003 27450903

[B27] Mansuri M. S. Singh M. Dwivedi M. Laddha N. C. Marfatia Y. S. Begum R. (2014). MicroRNA profiling reveals differentially expressed microRNA signatures from the skin of patients with nonsegmental vitiligo. Br. J. Dermatol. 171 (5), 1263–1267. 10.1111/bjd.13109 24814802

[B28] Mohammed G. F. Gomaa A. H. Al-Dhubaibi M. S. (2015). Highlights in pathogenesis of vitiligo. World J. Clin. Cases 3 (3), 221–230. 10.12998/wjcc.v3.i3.221 25789295 PMC4360494

[B29] Mueller D. W. Rehli M. Bosserhoff A. K. (2009). miRNA expression profiling in melanocytes and melanoma cell lines reveals miRNAs associated with formation and progression of malignant melanoma. J. Invest. Dermatol. 129 (7), 1740–1751. 10.1038/jid.2008.452 19212343

[B30] Parihar A. S. Tembhre M. K. Sharma V. K. Gupta S. Chattopadhyay P. Deepak K. K. (2020). Effect of narrowband ultraviolet B treatment on micro RNA expression in active non-segmental generalized vitiligo. Br. J. Dermatol. 183, 167–169. 10.1111/bjd.18890 31975367

[B31] Pauley K. M. Satoh M. Chan A. L. Bubb M. R. Reeves W. H. Chan E. K. (2008). Upregulated miR-146a expression in peripheral blood mononuclear cells from rheumatoid arthritis patients. Arthritis Res. Ther. 10 (4), R101. 10.1186/ar2493 18759964 PMC2575615

[B32] Rambow F. Bechadergue A. Saintigny G. Morizot F. Mahe C. Larue L. (2014). miR-330-5p targets tyrosinase and induces depigmentation. J. Invest. Dermatol. 134 (11), 2846–2849. 10.1038/jid.2014.231 24862846

[B33] Šahmatova L. Tankov S. Prans E. Aab A. Hermann H. Reemann P. (2016). MicroRNA-155 is dysregulated in the skin of patients with vitiligo and inhibits melanogenesis-associated genes in melanocytes and keratinocytes. Acta Derm. Venereol. 96 (6), 742–747. 10.2340/00015555-2394 26941046

[B34] Sahoo A. Lee B. Boniface K. Seneschal J. Sahoo S. K. Seki T. (2017). MicroRNA-211 regulates oxidative phosphorylation and energy metabolism in human vitiligo. J. Invest. Dermatol. 137 (9), 1965–1974. 10.1016/j.jid.2017.04.025 28502800 PMC6233982

[B35] Schallreuter K. U. Bahadoran P. Picardo M. Slominski A. Elassiuty Y. E. Kemp E. H. (2008). Vitiligo pathogenesis: Autoimmune disease, genetic defect, excessive reactive oxygen species, calcium imbalance, or what else? Exp. Dermatol. 17 (2), 139–140. discussion 141-60; discussion 141-60 (2008). 10.1111/j.1600-0625.2007.00666_1.x 18205713

[B36] Shang Z. Li H. (2017). Altered expression of four miRNA (miR-1238-3p, miR-202-3p, miR-630 and miR-766-3p) and their potential targets in peripheral blood from vitiligo patients. J. Dermatol. 44, 1138–1144. 10.1111/1346-8138.13886 28500632

[B37] Shi Q. Zhang W. Guo S. Jian Z. Li S. Li K. (2016). Oxidative stress-induced overexpression of miR-25: The mechanism underlying the degeneration of melanocytes in vitiligo. Cell Death Differ. 23 (3), 496–508. 10.1038/cdd.2015.117 26315342 PMC5072443

[B38] Shi Y. L. Weiland M. Lim H. W. Mi Q. S. Zhou L. (2014). Serum miRNA expression profiles change in autoimmune vitiligo in mice. Exp. Dermatol. 23 (2), 140–142. 10.1111/exd.12319 24401108

[B39] Su M. Miao F. Jiang S. Shi Y. Luo L. He X. (2020). Role of the p53TRPM1/miR211MMP9 axis in UVBinduced human melanocyte migration and its potential in repigmentation. Int. J. Mol. Med. 45 (4), 1017–1026. 10.3892/ijmm.2020.4478 31985026 PMC7053874

[B40] Su M. Yi H. He X. Luo L. Jiang S. Shi Y. (2019). miR-9 regulates melanocytes adhesion and migration during vitiligo repigmentation induced by UVB treatment. Exp. Cell Res. 384 (1), 111615. 10.1016/j.yexcr.2019.111615 31499059

[B41] Taieb A. Picardo M. (2009). Clinical practice. Vitiligo. N. Engl. J. Med. 360 (2), 160–169. 10.1056/NEJMcp0804388 19129529

[B42] Vachiramon V. Onprasert W. Harnchoowong S. Chanprapaph K. (2017). Prevalence and clinical characteristics of itch in vitiligo and its clinical significance. Biomed. Res. Int. 2017, 5617838. 10.1155/2017/5617838 28828385 PMC5554571

[B43] Vaish U. Kumar A. A. Varshney S. Ghosh S. Sengupta S. Sood C. (2019). Micro RNAs upregulated in Vitiligo skin play an important role in its aetiopathogenesis by altering TRP1 expression and keratinocyte-melanocytes cross-talk. Sci. Rep. 9 (1), 10079. 10.1038/s41598-019-46529-6 31300697 PMC6625998

[B44] Wang P. Li Y. Hong W. Zhen J. Ren J. Li Z. (2012). The changes of microRNA expression profiles and tyrosinase related proteins in MITF knocked down melanocytes. Mol. Biosyst. 8 (11), 2924–2931. 10.1039/c2mb25228g 22898827

[B45] Wang Y. Wang K. Liang J. Yang H. Dang N. Yang X. (2015). Differential expression analysis of miRNA in peripheral blood mononuclear cells of patients with non-segmental vitiligo. J. Dermatol. 42 (2), 193–197. 10.1111/1346-8138.12725 25495156

[B46] Whitton M. E. Pinart M. Batchelor J. Leonardi-Bee J. Gonzalez U. Jiyad Z. (2015). Interventions for vitiligo. Cochrane Database Syst. Rev. (2), CD003263. 10.1002/14651858.CD003263.pub5 25710794 PMC10887429

[B47] Whitton M. Pinart M. Batchelor J. M. Leonardi-Bee J. Gonzalez U. Jiyad Z. (2016). Evidence-based management of vitiligo: Summary of a cochrane systematic review. Br. J. Dermatol. 174 (5), 962–969. 10.1111/bjd.14356 26686510

[B48] Wu Q. Shi M. Meng W. Wang Y. Hui P. Ma J. (2019). Long noncoding RNA FOXD3-AS1 promotes colon adenocarcinoma progression and functions as a competing endogenous RNA to regulate SIRT1 by sponging miR-135a-5p. J. Cell. Physiol. 234 (12), 21889–21902. 10.1002/jcp.28752 31058315

[B49] Wu S. Yuan W. Shen Y. Lu X. Li Y. Tian T. (2017). The miR-608 rs4919510 polymorphism may modify cancer susceptibility based on type. Tumour Biol. 39 (6), 1010428317703819. 10.1177/1010428317703819 28653886

[B50] Xuan Y. Yang Y. Xiang L. Zhang C. (2022). The role of oxidative stress in the pathogenesis of vitiligo: A culprit for melanocyte death. Oxid. Med. Cell. Longev. 2022, 8498472. 10.1155/2022/8498472 35103096 PMC8800607

[B51] Zhang Z. Yang X. Liu O. Cao X. Tong J. Xie T. (2020). Differentially expressed microRNAs in peripheral blood mononuclear cells of non-segmental vitiligo and their clinical significance. J. Clin. Lab. Anal. 35, e23648. 10.1002/jcla.23648 33169883 PMC7891539

[B52] Zhao C. Wang D. Wang X. Mao Y. Xu Z. Sun Y. (2020). Down-regulation of exosomal miR-200c derived from keratinocytes in vitiligo lesions suppresses melanogenesis. J. Cell. Mol. Med. 24 (20), 12164–12175. 10.1111/jcmm.15864 32918341 PMC7579706

[B53] Zhao S. Fang F. Tang X. Dou J. Wang W. Zheng X. (2017). An in-depth analysis identifies two new independent signals in 11q23.3 associated with vitiligo in the Chinese Han population. J. Dermatol. Sci. 88 (1), 103. 10.1016/j.jdermsci.2017.05.001 28551095

